# Impact of Bone Mineral Density and Bone Structural Properties on Postmenopausal Women With Rheumatoid Arthritis in Japan: A Cross-Sectional Study

**DOI:** 10.7759/cureus.65056

**Published:** 2024-07-21

**Authors:** Yasuyuki Omichi, Fumio Shinomiya, Noriaki Mima, Jun Hirose, Tsutomu Enomoto, Tomohiro Goto, Shunji Nakano, Tetsuya Enishi, Masatoshi Morimoto, Koichi Sairyo

**Affiliations:** 1 Orthopedics, Tokushima Municipal Hospital, Tokushima, JPN; 2 Orthopedics, Institute of Biomedical Sciences, Tokushima University Graduate School, Tokushima, JPN; 3 Orthopedics, Mima Hospital, Yoshinogawa, JPN; 4 Rheumatology, Mima Hospital, Yoshinogawa, JPN; 5 Medical Informatics, Tokushima University Graduate School of Biomedical Sciences, Tokushima, JPN; 6 Radiology, Mima Hospital, Yoshinogawa, JPN; 7 Rehabilitation Medicine, Tokushima Municipal Hospital, Tokushima, JPN

**Keywords:** glucocorticoids, postmenopausal women, hip structure analysis, trabecular bone score, rheumatoid arthritis

## Abstract

Introduction

There has been no study on bone structural properties in postmenopausal women with rheumatoid arthritis (RA) in Japan. This study investigated bone mineral density (BMD) and bone structural properties in Japanese postmenopausal women with RA.

Methods

The study had a cross-sectional design and included 119 postmenopausal women aged 50-80 years with RA symptoms for more than five years. BMD, trabecular bone score (TBS), and results of hip structure analysis (HSA) were measured on dual-energy X-ray absorptiometry scans. The control group consisted of 288 women aged 50-80 years without RA. The RA group and control group using bisphosphonates were compared after propensity score matching for age, body mass index, and fracture history. Women in the RA group were also compared according to the use of glucocorticoids (GCs).

Results

After the propensity matching score, there were no other significant differences in BMD, TBS, and HSA parameters between the RA group and the control group. In the RA group, the TBS was lower in patients on GCs than those not on GCs (1.272 vs 1.313, p=0.008). There were no other significant differences in BMD and HSA parameters between patients in the RA group according to the use of GCs.

Conclusion

Although there were no differences in BMD, the TBS was lower in patients on GCs than those not on GCs in the RA group. It is thus important for physicians who administer GCs to treat patients with RA to be aware of not only BMD but also TBS.

## Introduction

Rheumatoid arthritis (RA) is a chronic inflammatory disease characterized by joint damage and systemic comorbidities. Osteoporosis is one of the major comorbidities in patients with RA. Autoimmune responses lead to increased osteoclastic bone resorption and impaired osteoblastic bone formation, an imbalance of which is responsible for bone loss in RA [1].

Postmenopausal osteoporosis is the most common type of osteoporosis, occurring in women as a consequence of estrogen deficiency [2]. Short-term and low-dose oral glucocorticoids (GCs) are effective in reducing the pain associated with RA. However, when used long-term and in high doses, GCs increase the risk of osteoporosis [3]. Aging and use of these agents are important risk factors for osteoporosis in patients with RA.

Osteoporosis is a skeletal disorder characterized by low bone mass and deterioration of bone quality with an increased risk of fracture [4]. Bone strength primarily reflects the integration of bone density and bone quality [4]. Bone density is expressed as bone mineral density (BMD), and bone quality refers to bone architecture, turnover, microfractures, and mineralization. Bone quality consists of material properties and structural properties [5], and trabecular bone score (TBS) and results of hip structure analysis (HSA), both of which can be obtained by dual-energy X-ray absorptiometry (DXA), are considered to reflect bone structural properties. The TBS is a gray-level textural index of trabecular bone structure that can be derived from a DXA image of the lumbar spine [6]. HSA is performed using DXA images of the hip and evaluates variables pertaining to proximal hip geometry, including the cross-sectional area (CSA), cross-sectional moment of inertia (CSMI), section modulus (Z), buckling ratio (BR), and cortical thickness (CT) [7]. Previous studies have reported that TBS and HSA are risk factors for fracture [6-8].

Osteoporosis is a major problem in postmenopausal patients with RA. There have been many reports on bone density but few on bone structural properties. Furthermore, there has been no study of bone structural properties in postmenopausal women with RA in Japan. The primary aim of this study was to investigate bone density and bone structural properties in postmenopausal women with RA in Japan. A secondary aim was to investigate the effects of GC use on bone density and structural properties.

## Materials and methods

Study design and participants

**Figure 1:**
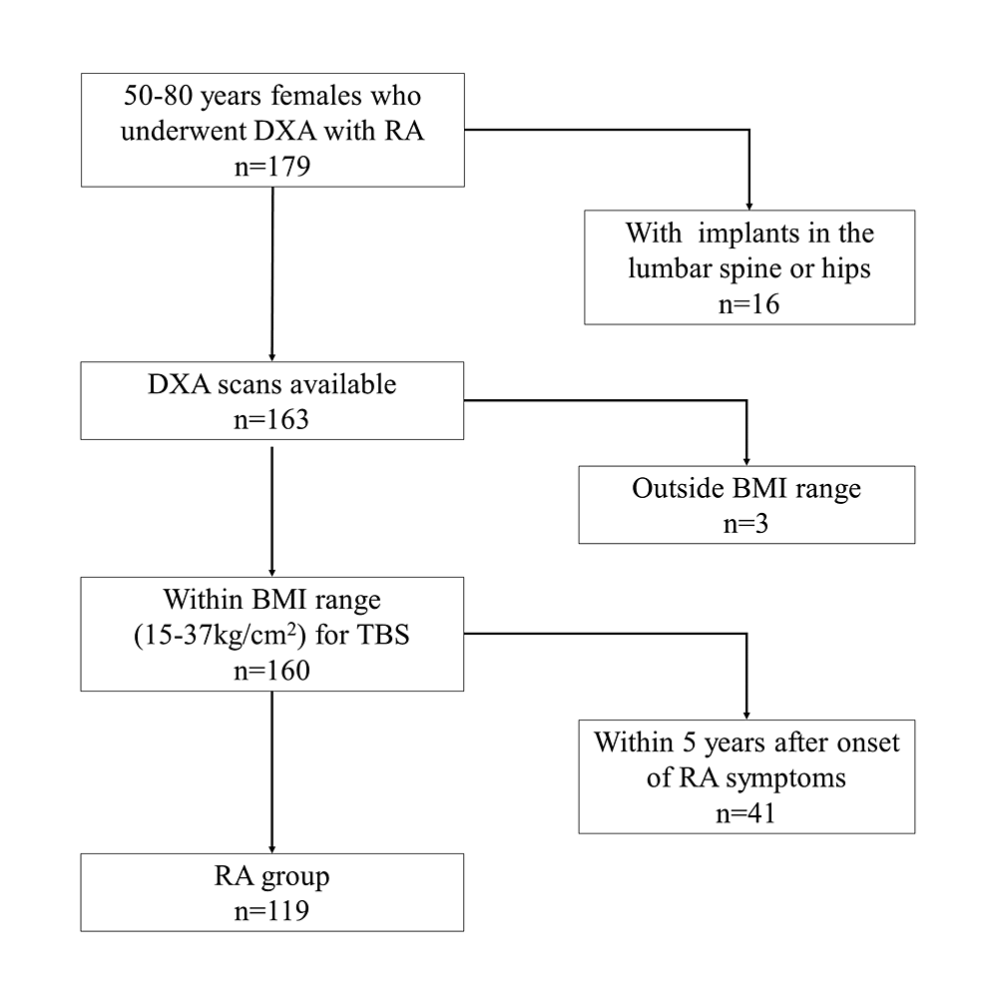
Flow chart showing the study enrollment process The data has been represented as n. BMI, body mass index; DXA, dual-energy X-ray absorptiometry; RA, rheumatoid arthritis; TBS, trabecular bone score

This cross-sectional study included 179 postmenopausal women (aged 50-80 years) who fulfilled the 2010 American College of Rheumatology/European League Against Rheumatism (EULAR) criteria for RA and underwent DXA at Mima Hospital between August 2019 and December 2022 [9]. Patients with implants in the lumbar spine or either hip, which could affect the accuracy of DXA measurements, were excluded. According to the manufacturer of the TBS software, the TBS is accurate for patients with a body mass index (BMI) in the range of 15-37. Therefore, patients with a BMI outside this range were also excluded, as were patients in whom the onset of RA symptoms was less than five years earlier, in order to evaluate conditions in the chronic phase of RA. Finally, the RA group included 119 patients (Figure 1). The control group consisted of 288 women aged 50-80 years who underwent DXA at Mima Hospital between August 2019 and December 2022. The control group included patients undergoing bone density screening, anti-osteoporosis treatment, and post-fracture rehabilitation. Patients using teriparatide, which is known to affect TBS and HSA, were excluded.

The following between-group comparisons were made. First, the demographic and clinical characteristics of the 119 patients in the RA group were compared with those of the 288 participants in the control group in the entire cohort. Second, 52 patients in the RA group who used bisphosphonates were compared with 52 participants in the control group who used bisphosphonates, after propensity score matching for age, BMI, and fracture history. Third, the 119 patients in the RA group were divided into two groups according to whether or not they were current users of GCs. Fourth, 46 patients in the RA group who used GCs were compared with 46 participants in the RA group who did not use GCs after propensity score matching for age, BMI, fracture history, and anti-osteoporosis agents.

Information on age, sex, height, weight, BMI, and past medical history (including type 2 diabetes mellitus, use of GCs, drug treatment for osteoporosis, and osteoporotic fracture) was obtained retrospectively from the medical records. In patients with RA, C-reactive protein (CRP), erythrocyte sedimentation rate (ESR), Disease Activity Score assessing 28 joints with CRP (DAS-28 CRP) and with ESR (DAS-28 ESR), rheumatoid factor (RF), and matrix metalloproteinase-3 were examined. The study was approved by our institutional ethics committee. Informed consent was obtained from all patients.

Bone mineral density

BMD was measured at the lumbar spine (L2-L4), femoral neck, and total femur using a DXA system (Horizon; Hologic, Inc., Marlborough, MA). Fractured vertebra or vertebra with a T-score > 1 with respect to the vertebra immediately above or below were excluded from analysis according to the exclusion criteria recommended by the International Society for Clinical Densitometry [10]. According to the World Health Organization criteria, osteoporosis is defined as a BMD that is ≤2.5 standard deviations (SDs) below the average value for healthy young adults (T-score −2.5 SD or less) and osteopenia as a BMD T-score between -1 and -2.5.

Trabecular bone score

The TBS is a textural index that evaluates gray-level pixel variations in DXA images of the lumbar spine and provides an indirect index of trabecular microarchitecture [11] and skeletal information that is not captured by standard BMD measurements. The TBS was measured using DXA software (TBS iNsight version 3.1; Medimaps Group SA, Geneva, Switzerland) and scored on the same anteroposterior DXA scan of the spine used to measure BMD. The TBS was calculated as the average score for the vertebral bodies at L2-L4. Vertebrae mentioned in the section on measurement of BMD was excluded from the analysis.

Hip structure analysis

The HSA program was used to assess hip strength indices by analyzing DXA images at the narrow neck (NN), intertrochanteric region (IT), and femoral shaft (FS) [7]. The HSA program locates these regions on the DXA proximal femoral bone mass images and derives the estimates from structural geometry. With these regions, the bone CSA (cm^2^), CSMI (cm^4^), Z (cm^3^), CT (cm), and buckling ratio (BR) were estimated from the bone profile. CSA is an indicator of resistance to loads directed along the bone axis. CSMI is relevant to bending in the plane of the DXA image. Z is an indicator of the ability of the bone to resist bending and torsion. CT is an estimate of mean cortical thickness and is calculated as the difference between the outer and inner diameters of the femoral cortex. BR is an index of susceptibility to local cortical buckling under compressive loads. The smaller the CSA, CSMI, Z, and CT, and the larger the BR, the greater the risk of fracture.

Statistical analysis

All data are expressed as the mean ± standard deviation. The characteristics of patients with and without vertebral fractures were compared using the Mann-Whitney U test and Pearson's chi-squared test as appropriate. All statistical analyses were performed using IBM SPSS Statistics for Windows, Version 29 (Released 2023; IBM Corp., Armonk, New York, United States). A p-value <0.05 was considered statistically significant.

## Results

Table 1 shows patient demographic and clinical data comparing the RA group and the control group in the entire cohort. The proportions of patients with a history of use of GCs and drugs for osteoporosis were significantly higher in the RA group (p<0.001). There were no significant differences in BMD at the lumbar spine, femoral neck, total hip, and TBS between the two groups. HSA revealed that CSA (FS), Z (FS), and CT (FS) were lower in the RA group (p=0.03, p=0.02, and p=0.02, respectively), while BR (FS) was higher in the RA group (p=0.03). There were no significant between-group differences in the other HSA parameters. There were 14.3% (17/119 patients) with clinical vertebral fractures (VF) and 2.5% patients (3/119 patients) with hip fractures in the RA group, whereas there were 24.0% (69/288 patients) with clinical vertebral fractures (VF) and 2.4% (7/288 patients) with hip fractures in the control group. The criterion for VF is grade 1 or higher by Genant’s semiquantitative (SQ) method using lateral X-ray images [12].

**Table 1:** Comparison of patient characteristics of the RA group and control group in the entire cohort ^a^Mann-Whitney U-test; Pearson's chi-squared test. The data has been represented as n, %, mean ± SD. A p-value <0.05 was considered statistically significant. BMD, bone mineral density; BMI, body mass index; BR, buckling ratio; CSA, cross-sectional area; CSMI, cross-sectional moment of inertia; CT, cortical thickness; DM, diabetes mellitus; FS, femoral shaft; GCs, glucocorticoids; IT, intertrochanteric region; NN, narrow neck; RA, rheumatoid arthritis; SERM, selective estrogen receptor modulator; TBS, trabecular bone score; Z, section modulus

Variable	RA group		Control group	p-value^a^
Number (n)		119	288	N/A
Age (years)		70.4 ± 5.5	71.5 ± 6.6	0.02
Height (cm)		151.2 ± 6.1	151.0 ± 6.6	0.76
Weight (kg)		51.4 ± 9.7	52.0 ± 9.4	0.59
BMI (kg/m^2^)		22.5 ± 3.9	22.8 ± 3.7	0.46
Duration of RA (years)		17.5 ± 10.0	0	<0.001
Type 2 DM (n, %)		4 (3.4%)	16 (5.6%)	0.35
Use of GCs (n, %)		48 (40.3%)	11 (3.8%)	<0.001
Vertebral fractures (n, %)		17 (14.3%)	69 (24.0%)	0.03
Hip fractures (n, %)		3 (2.5%)	7 (2.4%)	0.96
Osteoporosis drug treatment (n, %)		87 (73.1%)	109 (37.8%)	<0.001
Vitamin D supplementation (n, %)		32 (26.9%)	56 (19.4%)	0.10
SERM (n, %)		0 (0%)	15 (5.2%)	0.01
Bisphosphonate (n, %)		72 (60.5%)	65 (22.5%)	<0.001
Denosumab (n, %)		12 (10.1%)	5 (1.7%)	<0.001
BMD, lumbar spine (g/cm^2^)		0.79 ± 0.14	0.79 ± 0.14	0.74
BMD, femoral neck (g/cm^2^)		0.54 ± 0.09	0.54 ± 0.10	0.95
BMD, total hip (g/cm^2^)		0.66 ± 0.10	0.66 ± 0.13	0.41
TBS (L2–L4)		1.295 ± 0.075	1.314 ± 0.074	0.50
CSA (NN) (cm^2^)		1.83 ± 0.30	1.86 ± 0.35	0.41
CSMI (NN) (cm^4^)		1.41 ± 0.36	1.44 ± 0.39	0.62
Z (NN) (cm^3^)		0.78 ± 0.16	0.78 ± 0.18	0.88
CT (NN) (cm)		0.116 ± 0.021	0.115 ± 0.024	0.68
BR (NN) (cm^2^)		16.5 ± 4.4	16.8 ± 4.6	0.44
CSA (IT) (cm^2^)		3.36 ± 0.67	3.37 ± 0.64	0.86
CSMI (IT) (cm^4^)		8.98 ± 2.43	9.08 ± 2.65	0.79
Z (IT) (cm^3^)		2.83 ± 0.78	2.87 ± 0.72	0.21
CT (IT) (cm)		0.280 ± 0.054	0.283 ± 0.055	0.68
BR (IT) (cm^2^)		11.7 ± 2.7	11.6 ± 2.7	0.76
CSA (FS) (cm^2^)		2.86 ± 0.49	2.98 ± 0.51	0.03
CSMI (FS) (cm^4^)		2.15 ± 0.42	2.21 ± 0.55	0.24
Z (FS) (cm^3^)		1.44 ± 0.30	1.52 ± 0.32	0.02
CT (FS) (cm)		0.383 ± 0.080	0.402 ± 0.083	0.02
BR (FS) (cm^2^)		4.05 ± 1.22	3.78 ± 1.03	0.03

Table 2 compares the anthropometric, demographic, and clinical characteristics of the RA and control groups with the use of bisphosphonates, after propensity score matching for age, BMI, and fracture history. There were no significant between-group differences in the lumbar spine BMD, femoral neck BMD, total hip BMD, TBS, and the HSA parameters (CSA, CSMI, Z, CT, and BR).

**Table 2:** Comparison of patient characteristics of the RA group and control group by propensity score matching ^a^Mann-Whitney U-test; Pearson's chi-squared test. The data has been represented as n, %, mean ± SD. A p-value <0.05 was considered statistically significant. BMD, bone mineral density; BMI, body mass index; BR, buckling ratio; CSA, cross-sectional area; CSMI, cross-sectional moment of inertia; CT, cortical thickness; FS, femoral shaft; IT, intertrochanteric region; NN, narrow neck; RA, rheumatoid arthritis; TBS, trabecular bone score; Z, section modulus

Variable	RA group		Control group	value^a^
Number (n)		52	52	N/A
Age (years)		71.8 ± 4.7	71.8 ± 6.3	0.99
Height (cm)		150.8 ± 6.8	150.2 ± 6.6	0.66
Weight (kg)		50.3 ± 8.6	49.5 ± 9.6	0.66
BMI (kg/m^2^)		22.2 ± 4.0	22.0 ± 3.9	0.66
Duration of RA (years)		18.4 ± 11.6	0	<0.001
Bisphosphonate use (n, %)		52 (100%)	52 (100%)	N/A
BMD, lumbar spine (g/cm^2^)		0.77 ± 0.13	0.76 ± 0.12	0.74
BMD, femoral neck (g/cm^2^)		0.52 ± 0.08	0.53 ± 0.08	0.73
BMD, total hip (g/cm^2^)		0.65 ± 0.10	0.65 ± 0.10	0.82
TBS (L2–L4)		1.29 ± 0.08	1.30 ± 0.06	0.61
CSA (NN) (cm^2^)		1.77 ± 0.30	1.74 ± 0.26	0.60
CSMI (NN) (cm^4^)		1.41 ± 0.38	1.31 ± 0.30	0.13
Z (NN) (cm^3^)		0.77 ± 0.15	0.72 ± 0.13	0.08
CT (NN) (cm)		0.110 ± 0.020	0.110 ± 0.019	0.90
BR (NN) (cm^2^)		17.4 ± 4.2	17.3 ± 4.2	0.84
CSA (IT) (cm^2^)		3.26 ± 0.72	3.23 ± 0.53	0.80
CSMI (IT) (cm^4^)		8.50 ± 2.33	8.87 ± 2.54	0.43
Z (IT) (cm^3^)		2.72 ± 0.71	2.88 ± 0.59	0.21
CT (IT) (cm)		0.268 ± 0.050	0.271 ± 0.047	0.73
BR (IT) (cm^2^)		12.1 ± 2.8	11.9 ± 2.5	0.62
CSA (FS) (cm^2^)		2.77 ± 0.48	2.82 ± 0.40	0.59
CSMI (FS) (cm^4^)		2.09 ± 0.40	2.11 ± 0.56	0.84
Z (FS) (cm^3^)		1.41 ± 0.26	1.44 ± 0.30	0.68
CT (FS) (cm)		0.371 ± 0.071	0.375 ± 0.066	0.72
BR (FS) (cm^2^)		4.17 ± 1.29	4.04 ± 1.04	0.57

Table 3 shows the results when the RA group was divided according to whether the patients were on GCs (n=48) or not (n=71). The GC dose was ≤5 mg of prednisolone equivalent per day (mean dose 2.7 ± 1.3 mg) in all patients and the average duration of use was 10.3 ± 6.3 years. The TBS and RF were lower in patients in the RA group who were taking GCs (p=0.003 and p<0.001, respectively); there were no significant between-group differences in BMD at the lumbar spine, BMD at the femoral neck, BMD at the total hip, and the HSA parameters (CSA, CSMI, Z, CT, and BR).

**Table 3:** Comparison of RA patient characteristics according to the use of GCs The data has been represented as n, %, mean ± SD. A p-value <0.05 was considered statistically significant. BMD, bone mineral density; BMI, body mass index; BR, buckling ratio; CRP, C-reactive protein; CSA, cross-sectional area; CSMI, cross-sectional moment of inertia; CT, cortical thickness; DAS28, Disease Activity Score assessing 28 joints; DM, diabetes mellitus; ESR, erythrocyte sedimentation rate; FS, femoral shaft; GCs, glucocorticoids; IT, intertrochanteric region; MMP-3, matrix metalloproteinase-3; NN, narrow neck; RA, rheumatoid arthritis; RF, rheumatoid factor; TBS, trabecular bone score; Z, section modulus

Variable	RA with GCs		RA without GCs	p-value^a^
Number (n)		48	71	N/A
Age (years)		69.7 ± 5.8	70.9 ± 5.2	0.27
Height (cm))		150.1 ± 6.3	151.9 ± 6.2	0.21
Weight (kg)		52.0 ± 10.8	50.7 ± 9.0	0.63
BMI (kg/m^2^)		23.0 ± 4.5	21.9 ± 3.4	0.19
Duration of RA (years)		19.8 ± 9.2	16.1 ± 10.3	0.05
Duration of GCs (years)		10.3 ± 6.3	0	<0.001
GCS dose (mg)		2.7 ± 1.3	0	<0.001
Type 2 DM (n, %)		2 (4.2%)	2 (2.8%)	0.69
Vertebral fractures (n, %)		9 (18.9%)	8 (11.3%)	0.25
Hip fractures (n, %)		2 (4.2%)	1 (1.4%)	0.35
Osteoporosis drug treatment (n, %)		38 (79.2%)	49 (69.0%)	0.29
DAS-28 CRP		2.35 ± 1.06	2.27 ± 1.17	0.65
DAS-28 ESR		3.13 ± 1.26	3.09 ± 1.38	0.87
RF (IU/mL)		78.1 ± 103.2	97.2 ± 250.0	<0.001
MMP-3 (ng/mL)		115.6 ± 108.8	90.2 ± 149.8	0.32
BMD, lumbar spine (g/cm^2^)		0.80 ± 0.16	0.77 ± 0.13	0.33
BMD, femoral neck (g/cm^2^)		0.54 ± 0.10	0.55 ± 0.08	0.87
BMD, total hip (g/cm^2^)		0.64 ± 0.11	0.66 ± 0.09	0.25
TBS (L2–L4)		1.270 ± 0.081	1.311 ± 0.065	0.003
CSA (NN) (cm^2^)		1.81 ± 0.33	1.84 ± 0.28	0.71
CSMI (NN) (cm^4^)		1.39 ± 0.36	1.43 ± 0.36	0.53
Z (NN) (cm^3^)		0.76 ± 0.11	0.79 ± 0.15	0.27
CT (NN) (cm)		0.114 ± 0.024	0.116 ± 0.019	0.63
BR (NN) (cm^2^)		17.0 ± 5.4	16.1 ± 3.6	0.72
CSA (IT) (cm^2^)		3.27 ± 0.62	3.40 ± 0.67	0.28
CSMI (IT) (cm^4^)		8.82 ± 2.28	8.91 ± 2.31	0.83
Z (IT) (cm^3^)		2.85 ± 0.70	2.78 ± 0.78	0.91
CT (IT) (cm)		0.275 ± 0.057	0.281 ± 0.050	0.59
BR (IT) (cm^2^)		11.8 ± 2.9	11.6 ± 2.6	0.69
CSA (FS) (cm^2^)		2.79 ± 0.51	2.89 ± 0.48	0.28
CSMI (FS) (cm^4^)		2.10 ± 0.44	2.17 ± 0.41	0.34
Z (FS) (cm^3^)		1.42 ± 0.30	1.45 ± 0.30	0.58
CT (FS) (cm)		0.372 ± 0.082	0.387 ± 0.078	0.30
BR (FS) (cm^2^)		4.17 ± 1.30	3.99 ± 1.16	0.43

Table 4 compares the anthropometric, demographic, and clinical characteristics between the two groups in the RA group with and without the use of GCs after propensity score matching for age, BMI, fracture history, and anti-osteoporosis agents. Patients on GCs in the RA group had lower TBS than those not on GCs in the RA group (p=0.009). CT (FS) tended to be lower and BR (FS) tended to be higher in the RA group on GCs, but there were no significant differences between the two groups (p=0.10, p=0.09, respectively). There were no significant differences between patients in the RA group according to the use of GCs in the lumbar spine BMD, femoral neck BMD, total hip BMD, and the other HSA parameters.

**Table 4:** Comparison of patient characteristics in the RA group according to the use of GCs by propensity score matching ^a^Mann-Whitney U-test; Pearson's chi-squared test. The data has been represented as n, %, mean ± SD. A p-value <0.05 was considered statistically significant. BMD, bone mineral density; BMI, body mass index; BR, buckling ratio; CSA, cross-sectional area; CSMI, cross-sectional moment of inertia; CT, cortical thickness; FS, femoral shaft; GCs, glucocorticoids; IT, intertrochanteric region; NN, narrow neck; RA, rheumatoid arthritis; TBS, trabecular bone score; Z, section modulus

Variable	RA with GCs		RA without GCs	p-value^a^
Number (n)		46	46	N/A
Age (years)		69.6 ± 5.8	70.5 ± 5.6	0.42
Height (cm))		150.0 ± 6.3	151.5 ± 5.7	0.22
Weight (kg)		52.0 ± 10.8	51.7 ± 9.3	0.89
BMI (kg/m^2^)		23.1 ± 4.5	22.5 ± 3.5	0.46
Duration of RA (years)		20.1 ± 9.2	16.9 ± 11.0	0.13
Duration of GCs (years)		10.4 ± 6.3	0	<0.001
BMD, lumbar spine (g/cm^2^)		0.80 ± 0.15	0.78 ± 0.12	0.46
BMD, femoral neck (g/cm^2^)		0.54 ± 0.10	0.54 ± 0.07	0.97
BMD, total hip (g/cm^2^)		0.65 ± 0.11	0.67 ± 0.09	0.20
TBS (L2–L4)		1.272 ± 0.083	1.313 ± 0.064	0.009
CSA (NN) (cm^2^)		1.82 ± 0.33	1.86 ± 0.26	0.61
CSMI (NN) (cm^4^)		1.40 ± 0.36	1.48 ± 0.36	0.29
Z (NN) (cm^3^)		0.76 ± 0.17	0.80 ± 0.14	0.22
CT (NN) (cm)		0.115 ± 0.024	0.116 ± 0.018	0.77
BR (NN) (cm^2^)		17.0 ± 5.5	16.4 ± 4.0	0.59
CSA (IT) (cm^2^)		3.28 ± 0.62	3.42 ± 0.76	0.33
CSMI (IT) (cm^4^)		8.88 ± 2.23	8.95 ± 2.62	0.88
Z (IT) (cm^3^)		2.87 ± 0.69	2.79 ± 0.84	0.64
CT (IT) (cm)		0.277 ± 0.057	0.284 ± 0.054	0.55
BR (IT) (cm^2^)		11.8 ± 3.0	11.5 ± 2.8	0.66
CSA (FS) (cm^2^)		2.80 ± 0.51	2.93 ± 0.49	0.20
CSMI (FS) (cm^4^)		2.10 ± 0.43	2.14 ± 0.42	0.36
Z (FS) (cm^3^)		1.43 ± 0.31	1.45 ± 0.28	0.68
CT (FS) (cm)		0.373 ± 0.083	0.401 ± 0.076	0.10
BR (FS) (cm^2^)		4.17 ± 1.32	3.76 ± 0.87	0.09

## Discussion

This study investigated bone density and bone structural properties in Japanese postmenopausal women with RA. We found that the TBS was lower in patients on GCs in the RA group than those not on GCs in the RA group after propensity score matching for age, BMI, fracture history, and anti-osteoporosis agents. However, there were no significant differences in other parameters between patients in the RA group according to the use of GCs.

GCs are widely used in the treatment of RA, and their efficacy in reducing inflammation is widely recognized [13]. Many studies have shown that conventional synthetic disease-modifying antirheumatic drugs (csDMARDs) combined with low-dose GCs are more effective than csDMARDs alone in the short term in patients with early RA [13]. However, some studies have found no difference in efficacy between treatment with a combination of csDMARDs and GCs and treatment with csDMARDs alone in the long term [14]. In 2022, EULAR recommended that short-term GCs (for ≤3 months) be considered when initiating or changing csDMARDs but should be tapered and discontinued as rapidly as clinically feasible [15]. In 2021, the American College of Rheumatology conditionally recommended the initiation of csDMARDs without short-term GC therapy for DMARD-naive patients [16]. The consensus in the latest guidelines is that administration of GCs should be limited to low-dose and short-term use and that long-term use should be avoided in view of its toxicity.

There are numerous studies on the safety of GCs. Observational studies generally report significantly more adverse events in patients with RA taking GCs than in those not taking GCs in terms of cardiovascular events, infection, diabetes, osteoporosis, and low-dose GCs [17]. A recently updated review of randomized controlled trials concluded that the toxicity of low-dose GCs used for two years is mild and not significantly different from that of placebo [18]. The GLORIA study was a two-year pragmatic randomized controlled trial that assessed the safety and efficacy of GCs at a dose of 5 mg/day vs placebo when added to DMARDs in patients aged over 65 years with active RA [19]. Its main safety finding was that 60% of the GC group and 49% of the placebo group experienced a harmful outcome (adjusted relative risk 1.24), with the largest difference in the frequency of infections. The GLORIA study also showed that spinal bone density decreased by about 1% in the GC group but increased by 3% in the placebo group; the difference was statistically significant [20]. However, bisphosphonates and other anti-osteoporosis medications are effective in preventing GC-induced fragility fractures [21]. In our study, 80% of patients on GCs in the RA group received anti-osteoporosis treatment, and there was no significant difference in BMD or incidence of fragility fractures in the RA group according to whether or not patients were taking GCs. Our failure to identify any specific risk factors for fragility fractures of the vertebra may have been due in part to the use of anti-osteoporosis medications.

Previous studies have shown that postmenopausal women with RA patients have lower TBS values [22]. Furthermore, the TBS has been reported to be useful for assessing the fracture risk in patients with RA [23]. A study of 279 postmenopausal women with RA and over the age of 50 years found that TBS was lower in patients with VF than in those without VF despite similar BMD at the lumbar spine [23].

There have been reports on the impact of GC therapy on TBS [24,25]. Paggiosi et al. reported that TBS was lower in postmenopausal women treated with GCs (mean dose 7.2 ± 3.2 mg for a mean duration of 9.2 ± 10.8 years) than in untreated women despite there being no difference in BMD at the lumbar spine between the two groups [24]. Corrado et al. evaluated 47 subjects with recent-onset RA and found an inverse relationship between the TBS and the cumulative GC dose in their high-dose GC group but not in their low-dose GC group [25]. In our study, patients with RA who were on GCs had lower TBS, suggesting that the cumulative GC dose contributes to lower TBS and deterioration of bone quality. The TBS could be a useful tool for assessing the adverse effects of GCs on bone microarchitecture.

There is one longitudinal study of HSA in patients with RA [26]. Wright et al. reported that the RA group (n=78) had a significantly lower outer diameter, CSA, and Z at the femoral neck regions than the non-arthritic control group (n=4779) in the longitudinal models [26]. They also reported that no significant associations were seen at the intertrochanteric or femoral shaft regions, and the association was not modified by age, ethnicity, glucocorticoid use, or time [26]. In our study, CT (FS) tended to be lower (p=0.10) and BR (FS) tended to be higher (p=0.09) in the RA group on GCs than those in the RA group, but not significantly, therefore which is also consistent with Wright's report. Utility of HSA is still limited by difficulties in interpreting the structural parameters and by insufficient evidence from clinical practice settings regarding fracture prediction [27]. Beck, who developed the HSA programs, reported that BR should generally be relevant only for femoral neck and intertrochanteric regions and is unlikely to have any strength effect at the femur shaft [7]. Beck also reported that the HSA program generates a number of intermediate parameters, which may be useful but are not themselves strength indices, for example, average cortical thickness [7]. In our study, there was a trend toward differences in CT(FS) and BR(FS) in the RA group on GCs, but these parameters themselves may not have clinical significance. The latest several reviews pointed out the difficulty in interpreting the structural parameters and insufficient evidence from clinical practice settings regarding fracture prediction [28]. Therefore, ISCD does not recommend HSA parameters for assessing hip fracture risk [29].

The primary strength of this study is that it is the first to investigate bone structural properties in Japanese postmenopausal patients with RA. A meta-analysis of 14 cohort studies showed that the mean TBS for postmenopausal women varied according to ethnicity [30]. This is the first report showing that in Asian people, as well as in other racial groups, GC use in RA patients results in decreased TBS.

This study also has several limitations. First, it had a retrospective cross-sectional design and was performed at a single center. The control group included patients undergoing bone density screening, anti-osteoporosis treatment, and post-fracture rehabilitation, which may be biased. The rate of vertebral fractures is higher in the control group (24.0% vs 14.3%), and the control group might be at higher risk for osteoporosis. Therefore, it is possible that there was no difference in BMD between the RA group and the control group. Second, patients who were using anti-osteoporosis medication were not excluded. However, the effectiveness of these medications in patients with RA and osteoporosis is well known, and controlling for anti-osteoporosis drugs would have been unrealistic. In addition, anti-resorptive therapy has little effect on TBS [29]. We compared the RA group to the control group using only bisphosphonates for the treatment of osteoporosis (Table 2). We believe that the effect of anti-osteoporosis treatment was minimized by aligning the patient's osteoporosis treatment background. Third, there is no data on the material properties of bone within bone quality. Fourth, the number of subjects was relatively small, which might have prevented significant differences in HSA parameters in the RA group.

## Conclusions

This is the first study to investigate BMD and bone structural properties in postmenopausal women with RA in Japan. We found that TBS was lower in patients in the RA group on GCs than those not on GCs, even though there were no differences in BMD between the two groups after propensity score matching for age, BMI, fracture history, and anti-osteoporosis agents. Therefore, it is important for physicians who administer GCs to treat patients with RA to be aware of not only BMD but also TBS. Further studies are needed to determine whether HSA is worthwhile in these patients.
